# Is duration of residence a proxy for acculturation? The case of health risk behaviors among international immigrants

**DOI:** 10.1177/14034948231199534

**Published:** 2023-09-18

**Authors:** Sol P. Juárez, Helena Honkaniemi, Nina-Katri Gustafsson, Lisa Berg

**Affiliations:** 1 Department of Public Health Sciences, Stockholm University, Sweden; 2 Centre for Health Equity Studies (CHESS), Stockholm University/Karolinska Institutet, Sweden

**Keywords:** Emigrants and immigrants, health risk behaviors, acculturation, socioeconomic factors

## Abstract

**Aims:**

Among international immigrants, health changes by duration of residence are commonly interpreted as an expression of acculturation to the receiving country context. This study compares changes in immigrants’ health risk behaviors by duration of residence to changes by acculturation levels, in order to assess whether duration of residence can be regarded as a proxy for acculturation.

**Methods:**

Using data from a previous systematic review, we identified 17 quantitative studies examining changes in alcohol, tobacco and drug use, physical inactivity, and diet by both duration of residence and acculturation level in the same population. We compared the directionality and consistency of these associations through tabulation and vote counting.

**Results:**

The majority of studies reported no or inconsistent changes in health risk behaviors by duration of residence versus by acculturation, including with opposite directionality. Four studies reported significant estimates with consistent directionality, while five reported consistent, non-significant estimates.

**Conclusions:**

**Our findings suggest that duration of residence should not be used as a proxy for acculturation when studying health risk behaviors among immigrants. Researchers should consider additional time-dependent factors to explain behavioral changes by duration of residence**.

## Introduction

Various acculturation measures have been designed over the past decades to categorize immigrants and their descendants by their level of belonging or proximity to the receiving country context, in terms of their ethnic identity, language proficiency, and cultural orientation, among other dimensions [1]. In turn, these indicators of acculturation have been used to explain immigrants’ behavioral changes [2]. Multiple reviews have shown that with greater acculturation, immigrants tend to adopt various health risk behaviors including alcohol, tobacco and drug use, physical inactivity, and poor diet (the so-called acculturation paradox), often converging with behavioral levels of the native population in the receiving country [3–6].

The cultural interpretation of immigrants’ behavioral changes is not limited to studies utilizing acculturation measures specifically. A recent systematic review on immigrants’ health risk behaviors highlighted that behavioral changes by duration of residence (DoR) are also commonly attributed to acculturation processes [7]. In other words, DoR is often used as a proxy for acculturation, based on the assumption that immigrants tend to experience greater levels of identification with native norms and practices of the receiving country over time. However, this has been contested from a health equity perspective, given that immigrants tend to adopt health risk behaviors that are already unequally distributed *within* the native population (so-called unequal assimilation) [8]. In fact, this unequal distribution of risk behaviors is key for understanding the reproduction of health inequalities in society.

Beyond acculturation, DoR is regarded as a valuable public health measure to monitor immigrants’ health over time, reflecting other processes such as cumulative experiences of socioeconomic inequalities and discrimination [9]. Yet, without empirical evidence, the assumption that DoR directly corresponds to acculturation limits consideration of these alternative time-dependent processes underlying immigrant health changes [1,7,9]. In order to assess the applicability of DoR as a proxy for acculturation, the aim of this study was thus to investigate how changes in immigrants’ health risk behaviors by DoR compared with changes by their acculturation levels.

## Methods

This study is based on material from our previously published systematic review on international immigrants’ health risk behaviors by DoR [7,10]. In the review, the PubMed/MEDLINE, Web of Science and ProQuest databases were searched for English-language peer-reviewed articles published from 1 January 2000 to 31 December 2019, which quantitatively examined changes in health risk behaviors (i.e., alcohol, tobacco and drug use, physical inactivity, and diet) by DoR among international first-generation immigrants. For the current study, we included all articles from the review that additionally evaluated changes in risk behaviors by acculturation level. Data was extracted on receiving country, immigrant population, health risk behavior, and on DoR and acculturation measures. Tabulation and vote counting were applied to compare the directionality (i.e., increased, null, or decreased) and consistency of associations between DoR and risk behaviors, on the one hand, and acculturation and risk behaviors, on the other. No ethical approval was required for assessment of the secondary data.

## Results

Of the 123 articles included in the original systematic review [7], 17 articles [11–27] examined one or more health risk behaviors among immigrants by both DoR and acculturation level (for study descriptions and comparisons, see Table I). All studies were cross-sectional, from the USA (*n* = 10) or Europe (*n* = 7), and examined alcohol (*n* = 7), tobacco (*n* = 4), drug use (*n* = 1), physical activity (*n* = 5), and/or diet (*n* = 3). DoR was measured categorically (*n* = 11) and/or continuously (*n* = 7). Acculturation indices captured one or more dimensions relating to social networks/interactions (*n* = 9), cultural orientation/competence (*n* = 8), language use (*n* = 6), ethnic identity (*n* = 3), sense of belonging (*n* = ), and/or cultural behaviors/preferences (*n* = 1).

**Table I.** Overview of the 17 studies included in the literature review and the consistency of associations between immigrant health risk behaviors and measures of duration of residence versus acculturation.

Study	Country	Immigrant population	Outcome(s)	Directionality of associations: duration of residence measure	Directionality of associations: acculturation measure	Consistency between results in relation to duration of residence versus acculturation (Yes/No)*
Addo et al. [11]	Netherlands, Germany, UK	Ghanaians	Alcohol use	Categorical: 1–5, 5–9 years vs. 10+ years (Ref)	Acculturation items: ethnic identity, cultural orientation, social networks	Yes (men/women: ethnic identity, cultural orientation) No (men/women: social networks)
		Men	Alcohol use	Increases with longer residence	Increases with more acculturation (*ethnic identity*) Does not significantly change with more acculturation (*cultural orientation*: non-significant increase; *social networks*: null)	Yes (increase) Yes (increase vs. non-significant increase) No (increase vs. null)
		Women	Alcohol use	Increases with longer residence	Does not significantly change with more acculturation (*ethnic identity, cultural orientation*: non-significant increase; *social networks*: non-significant decrease)	Yes (increase vs. non-significant increase) No (increase vs. non-significant decrease)
Amundsen [12]	Norway	Iranians, Pakistanis, Turkish	Alcohol use	Continuous: years	Acculturation items: home and host culture competence, social interaction	No (All, Pakistani and Turkish) Yes (Iranian)
		All	Alcohol use	Decreases with longer residence	Increases with more acculturation (*home culture competence (low), host culture competence, social interaction*)	No (decrease vs. increase)
		Iranian	Alcohol use	Increases with longer residence	Does not significantly change with more acculturation (*home culture competence (low), host culture competence*: non-significant increase) Increases with more acculturation (*social interaction*)	Yes (increase vs. increase/non-significant increase)
		Pakistani	Alcohol use	Does not significantly change with longer residence (null)	Increases with more acculturation (*home culture competence (low), host culture competence*) Does not significantly change with more acculturation (*social interaction*: non-significant increase)	No (null vs. increase/non-significant increase)
		Turkish	Alcohol use	Does not significantly change with longer residence (null)	Increases with more acculturation (*host culture competence, social interaction*) Does not significantly change with more acculturation (*home culture competence (low)*: non-significant increase)	No (null vs. increase/non-significant increase)
Brathwaite et al. [13]	Netherlands, Germany, UK	Ghanaians (men)	Smoking	Continuous: years Categorical: <10 years (Ref) vs. 10–19, 20–29, 30+ years	Acculturation items: ethnic identity, cultural orientation, social networks (all categorized as assimilation, separation and marginalization vs. integration)	Yes (ethnic identity, cultural orientation) No (social networks)
		All	Smoking	Does not significantly change with longer residence (*continuous*: null; *30+ years’ category*: non-significant increase)	Does not significantly change with more acculturation (*ethnic identity*: non-significant increase) Increases with more acculturation (*cultural orientation*) Does not significantly change with more acculturation (*social networks*: non-significant decrease)	Yes (non-significant increase) Yes (non-significant increase vs. increase) No (non-significant increase vs. non-significant decrease)
Borges et al. [14]	USA	Mexicans	Alcohol use Subclinical alcohol dependence Alcohol dependence syndrome	Categorical: 0–6 years (Ref) vs. 7–12, 12–20, 20–55 years, US-born	Acculturation scale: language use, social networks	No (men; women: subclinical alcohol dependence) Yes (women: alcohol dependence syndrome)
		All	Subclinical alcohol dependence	Does not significantly change with longer residence (null)	Does not significantly change with more acculturation (null)	Yes (null)
		All	Alcohol dependence syndrome	Does not significantly change with longer residence (non-significant increase)	Does not significantly change with more acculturation (non-significant decrease)	No (non-significant increase vs. decrease)
		Men	Subclinical alcohol dependence	Does not significantly change with longer residence (non-significant increase)	Does not significantly change with more acculturation (non-significant decrease)	No (non-significant increase vs. decrease)
		Men	Alcohol dependence syndrome	Does not significantly change with longer residence (non-significant increase)	Does not significantly change with more acculturation (non-significant decrease)	No (non-significant increase vs. decrease)
		Women	Subclinical alcohol dependence	Does not significantly change with longer residence (non-significant decrease)	Increases with more acculturation	No (non-significant decrease vs. increase)
		Women	Alcohol dependence syndrome	Does not significantly change with longer residence (non-significant increase)	Does not significantly change with more acculturation (non-significant increase)	Yes (non-significant increase)
Canfield et al. [15]	UK	Brazilians	Alcohol use Smoking Drug use	Continuous: years	Acculturation, Habits and Interests Multicultural Scale for Adolescents: social networks, behaviors and preferences Assimilation (not considering integration, separation and marginalization)	Yes (alcohol use, regular drinking) No (alcohol use, binge drinking) No (smoking) No (drug use)
		All	Alcohol use	Regular drinking risk does not significantly change with longer residence (null) Binge drinking risk does not significantly change with longer residence (null)	Regular drinking risk does not significantly change with more acculturation (null) Binge drinking risk does not significantly change with more acculturation (non-significant increase)	Yes (null) No (null vs. non-significant increase)
		All	Smoking	Does not significantly change with longer residence (null)	Does not significantly change with more acculturation (non-significant increase)	No (null vs. non-significant increase)
		All	Drug use	Decreases with longer residence	Does not significantly change with more acculturation (null)	No (decrease vs. null)
Choi et al. [16]	USA	Korean (women)	Physical inactivity	Continuous: years	Vancouver Index of Acculturation: orientation toward mainstream and heritage traditions	No
		All	Physical inactivity (leisure-time)	Increases with longer residence	Does not significantly change with more acculturation (null)	No (increase vs. null)
Commodore-Mensah et al. [17]	USA	Ghanaians, Nigerians	Physical inactivity	Categorical: <10 vs. 10+ years	Modified Psychological Acculturation Scale: sense of psychological attachment to and belonging within own and host culture, social network	No
		Men	Physical inactivity (leisure-time & work)	Does not significantly change with longer residence (non-significant decrease)	Does not significantly change with more acculturation (null)	No (non-significant decrease vs. null)
		Women	Physical inactivity (leisure-time & work)	*Same as above*	*Same as above*	*Same as above*
Constantine et al. [18]	USA	Latinos, Southeast Asians	Smoking	Continuous: proportion of life lived in USA	Acculturation items: fluency with home country culture, comparison with host country natives, ethnicity of social network	Yes (men: fluency with home country culture) No (men: comparison with host country natives, ethnicity of social network; women: fluency with home country culture, comparison with host country culture, ethnicity of social network)
		Men	Smoking	Increases with longer residence	Increases with more acculturation (fluency with home country culture (low)) Does not significantly change with more acculturation (*comparison with host country natives, ethnicity of social network)*	Yes (increase) No *NB: Non-significant estimates not reported, undetermined if null.*
		Women	Smoking	Increases with longer residence	Does not significantly change with more acculturation (*fluency with home country culture (low), ethnicity of social network*) Decreases with more acculturation (*comparison with host country natives*)	No No (increases vs. decreases) *NB: Non-significant estimates not reported, undetermined if null.*
Evenson et al. [19]	USA	Latinas	Physical inactivity	Categorical: <3 vs. ⩾3 years	Latino English language acculturation scale	Yes
		All	Physical inactivity (leisure-time)	Does not significantly change with longer residence (non-significant increase)	Increases with more acculturation	Yes (non-significant increase vs. increase)
		All	Physical inactivity (work)	Does not significantly change with longer residence (non-significant decrease)	Does not significantly change with more acculturation (non-significant decrease)	Yes (non-significant decrease)
		Mexican	Physical inactivity (leisure-time & work)	*Same as above*	*Same as above*	*Same as above*
Ham et al. [20]	USA	Latinos	Physical inactivity	Categorical: 10–14 vs. 15+ years	Latino English language acculturation scale	Yes
		All	Physical inactivity (daily)	Does not significantly change with longer residence (null)	Does not significantly change with more acculturation (null)	Yes (null)
Koca and Lapa [21]	Germany & England	Turkish	Physical inactivity	Categorical: Germany: ⩽25 vs. >26 years; England: ⩽15 vs. >16 years	Acculturation item: language proficiency	Yes
	Germany	Turkish	Physical inactivity	Does not significantly change with longer residence (null)	Does not significantly change with more acculturation (null)	Yes (null)
	England	Turkish	Physical inactivity	*Same as above*	*Same as above*	*Same as above*
Nicolau et al. [22]	Netherlands	Surinamese South Asians, Surinamese Afro-Caribbean	Diet	Categorical: 0–17, 18–26, 27+ years	Acculturation item: social contacts	Yes
		Afro-Caribbean (men)	Diet quality	Does not significantly change with longer residence (null)	Does not significantly change with more acculturation (null)	Yes (null)
		South Asian (men)	Diet quality	*Same as above*	*Same as above*	*Same as above*
		Afro-Caribbean (women)	Diet quality	*Same as above*	*Same as above*	*Same as above*
		South Asian (women)	Diet quality	*Same as above*	*Same as above*	*Same as above*
Osei-Kwasi et al. [23]	Netherlands, Germany, UK	Ghanaians	Alcohol use	Categorical: 0–10 years (Ref) vs. 11−20, 20+ years	Acculturation items: ethnic identity, cultural orientation, social networks	No
		All	Alcohol intake frequency	Increases with longer residence	Does not significantly change with more acculturation (null)	No (increase vs. null)
Park et al. [24]	USA	Asians	Alcohol use	Categorical: 0–10 years (Ref) vs. 11–20, >20 years	Acculturation item: language proficiency	Yes (all Asian) No (Chinese) Yes (Filipino) No (Vietnamese)
		All	Frequency/quantity of alcohol use	Increases with longer residence	Increases with more acculturation	Yes (increase)
		Chinese	Frequency/quantity of alcohol use	Does not significantly change with longer residence (null)	Increases with more acculturation	No (null vs. increase)
		Filipino	Frequency/quantity of alcohol use	Increases with longer residence	Increases with more acculturation	Yes (increase)
		Vietnamese	Frequency/quantity of alcohol use	Does not significantly change with longer residence (null)	Increases with more acculturation	No (null vs. increase)
Tseng and Fang [25]	USA	Chinese (women)	Diet	Continuous: years	Abridged version of General Ethnicity Questionnaire-American Version: cultural orientation	Yes
		All	Caloric intake	Does not significantly change with longer residence (null)	Does not significantly change with more acculturation (null)	Yes (null)
Tseng et al. [26]	USA	Chinese (women)	Diet	Continuous: years	Abridged version of General Ethnicity Questionnaire-American Version: cultural orientation	Yes
		All	Caloric intake	Does not significantly change with longer residence (non-significant increase)	Does not significantly change with more acculturation (non-significant increase)	Yes (non-significant increase)
Wong et al. [27]	USA	Southeast Asians	Alcohol use Smoking	Categorical: 1–19 vs. 20+ years	Acculturation item: language use	No (alcohol use, beer) Yes (alcohol use, other alcohol) Yes (smoking)
		All	Alcohol use	(Beer use) does not significantly change with longer residence (non-significant decrease) (Other alcohol use) does not significantly change with longer residence (non-significant increase)	Increases with more acculturation	No (for beer; non-significant decrease vs. increase) Yes (for other alcohol; non-significant increase vs. increase)
		All	Smoking	Does not significantly change with longer residence (non-significant increase)	Does not significantly change with more acculturation (non-significant increase)	Yes (non-significant increase)

* “Yes” indicates that findings in the same study/population are consistent (i.e., concordance in directionality) and “No” that findings are not consistent (i.e., discrepancies in directionality).

Eleven of the 17 articles reported *inconsistent* estimates with regard to the directionality of findings for health risk behaviors by DoR versus by acculturation, either with opposite directionality, irrespective of significance [11–14,18,27], or with one null finding [11,12,15–18,23,24]. This included studies with composite and specific measures of acculturation (i.e., social networks) [11,13,18]. For *consistent* findings, only four articles reported consistent, significant estimates [11,12,18,24], for men [11,18] or specific immigrant groups [12,24]. Five studies reported non-significant estimates of the same direction [13,14,19,26,27], and another six studies consistently null findings [14,15,20–22,25]. Consistent findings were found between DoR and composite or specific acculturation measures of cultural orientation/competence [11,13,18,25,26], ethnic identity [11,13], and language [19,20,21,24,27].

Findings from studies on alcohol use by DoR versus acculturation varied, demonstrating null or inconsistent changes [11,12,14,15,23,24,27] or consistent changes in the same direction [11,12, 14,24,27]. Studies on smoking [13,15,18], drug use [15], physical inactivity [16,20,21], and diet [22,25] also generally showed null or inconsistent findings, with some exceptions indicating same directionality [18,19,26,27].

## Discussion

Most of the 17 reviewed studies showed discrepancies between international immigrants’ changes in health risk behaviors by DoR versus by acculturation levels. Only four studies demonstrated estimates with consistent directionality, while others reported inconsistently null findings or findings with opposite directionality. We noted important differences by specific measures of acculturation and population groups (i.e., by gender and origin). These findings challenge the use of DoR as a proxy for acculturation in immigrant health research.

The inconsistencies observed in this study support the need to consider alternative time-dependent explanations of immigrant health changes beyond acculturation, such as cumulative experiences of socioeconomic disadvantage and discrimination. These can influence the adoption of unhealthy habits over time as a means of stress relief [9]. Moreover, the fact that these health risk behaviors are socially patterned within the receiving society may suggest that their adoption among immigrants is also linked to social inequalities [8].

In addition to the inconsistencies *between* DoR and acculturation measures, our findings did not support the existence of a universal *acculturation* pathway—not all studies found that immigrants adopted risk behaviors with increased acculturation, irrespective of DoR. This contradiction with previous findings could be attributable to a select sample of acculturation studies (i.e., with DoR measures), although it is more likely that acculturation processes vary across immigrant groups. Moreover, we found no universal pattern of behavioral change by longer DoR, supporting the results of our previous systematic review [7].

This is the first effort to compare changes in health risk behaviors by DoR to those by acculturation *within the same populations*. Comparisons were challenged by a lack of between-study consensus on definitions and operationalizations of DoR and acculturation. The general reliance on statistically significant results, which are strongly dependent on how DoR and acculturation are measured, and the varied model specifications between measures and across studies, posed additional challenges for comparison. Yet, we found evidence of inconsistent results between measures that are unlikely to be influenced by the above-mentioned limitations.

To date, public health researchers have primarily relied on theories of acculturation to interpret changes in immigrants’ health behaviors [7]. This study suggests that the appropriateness of DoR as a proxy for acculturation needs to be carefully re-examined, with further consideration of the role of social factors alongside acculturative processes in the adoption of these behaviors [1,9]. Our findings support the need to detach evidence on DoR from the acculturation framework and consider alternative explanations for immigrants’ health changes.
